# Predictive Value of Preoperative Left Atrial Coupling Indices for Postoperative Atrial Fibrillation After Isolated CABG

**DOI:** 10.3390/medicina62020353

**Published:** 2026-02-10

**Authors:** Hasan Ali Sinoplu, Atilla Koyuncu, Cennet Yıldız, Fatma Nihan Turhan Çağlar, Dilay Karabulut, Hasan Toz, Mehmet Pişirici, Büşra Mavi, Atakan Arpaç, Alparslan Şahin

**Affiliations:** 1 Department of Cardiology, Bakırköy Dr. Sadi Konuk Training and Research Hospital, 34147 Istanbul, Turkey; atikoyuncu@gmail.com (A.K.); cennet_yildiz@live.com (C.Y.); nhnturhan@gmail.com (F.N.T.Ç.); dilay_karakozak@hotmail.com (D.K.); pisiricimehmet@gmail.com (M.P.); atakan.arpac@gmail.com (A.A.); dralpsahin@gmail.com (A.Ş.); 2 Department of Cardiovascular Surgery, Bakırköy Dr. Sadi Konuk Training and Research Hospital, 34147 Istanbul, Turkey; tozhasan@hotmail.com; 3 Department of Cardiology, Bahçelievler Government Hospital, 34192 Istanbul, Turkey; mavi.busra.93@gmail.com

**Keywords:** atrial fibrillation, POAF, LACI, echocardiography

## Abstract

*Background and Objectives:* Postoperative atrial fibrillation (POAF) is the most common arrhythmia after coronary artery bypass grafting (CABG) and is linked to adverse outcomes. This study evaluated the predictive value of the Left Atrial Coupling Index (LACI) for POAF and compared two calculation methods, LACI_1_ and the novel LACI_2_. *Materials and Methods:* This prospective study included 130 patients undergoing isolated CABG between January 2022 and June 2023. Preoperative echocardiography was performed to calculate conventional parameters and LACI values: LACI_1_ = LAVI/TDI-septal a′ and LACI_2_ = LAVI/min (TDI-septal a′, TDI-lateral a′). Patients were classified into POAF (+) and POAF (−) groups. Clinical, echocardiographic, and outcome data were compared. Logistic regression and receiver operating characteristic (ROC) analyses were performed. *Results:* POAF occurred in 59 patients (45.4%). Those with POAF were older, had more diabetes mellitus(DM), hypertension(HT), and higher EuroSCORE II values (all *p* < 0.05). POAF was associated with longer hospitalization and higher in-hospital mortality. Both LACI_1_ (4.21 ± 2.62 vs. 2.94 ± 1.02, *p* < 0.001) and LACI_2_ (4.27 ± 2.60 vs. 2.96 ± 1.02, *p* < 0.001) were significantly higher in the POAF group. In multivariate analysis, LACI_1_ (OR 1.45, *p* = 0.020) and LACI_2_ (OR 1.50, *p* = 0.004) remained independent predictors. ROC analysis showed numerically higher discriminatory performance for LACI_2_ (AUC = 0.690, specificity 74.5%) compared with LACI_1_ (AUC = 0.677, specificity 67.6%). *Conclusions:* LACI is an independent predictor of POAF after CABG. The novel LACI_2_ demonstrated numerically higher predictive performance compared with LACI_1_ and may improve preoperative risk stratification and guide preventive strategies.

## 1. Introduction

Atrial fibrillation (AF) continues to maintain its importance in cardiology practice with a steadily increasing incidence each year. Particularly in the elderly population, AF has been reported to develop in approximately 1 in 3 individuals [1]. In younger patients, long-term intensive endurance sports have been reported as an independent risk factor for the development of AF [2]. In addition, alcohol consumption, cigarette smoking, hypertension (HT), diabetes mellitus (DM), hyperthyroidism, surgery, infection, acute myocardial infarction, acute pericardial disease, pulmonary embolism, or other acute pulmonary disease are well-established risk factors for the development of AF [3,4,5,6]. Cardiomyopathies are recognized as one of the causes of secondary AF [7]. Postoperative atrial fibrillation (POAF) is one of the most common forms of secondary AF [7,8]. It is an arrhythmia associated with increased morbidity and mortality following both cardiac and non-cardiac surgical procedures [9].

Coronary artery bypass grafting (CABG) is a well-established and widely utilized surgical strategy in the management of coronary artery disease. POAF is the most common arrhythmia following CABG [10] and generally occurs within the first 2 to 4 days after surgery [11]. The incidence of POAF following CABG is reported to range between 25% and 50% [11], and this arrhythmia is associated with serious complications such as prolonged hospitalization, heart failure, stroke, and increased mortality [10,12]. Therefore, preoperative identification of patients at high risk for POAF is essential to improve clinical outcomes and reduce postoperative complications.

The structural and functional integrity of the left atrium (LA) plays a pivotal role in the pathogenesis of AF [11,13,14]. Although traditional echocardiographic parameters—particularly the Left Atrial Volume Index (LAVI)—are useful for evaluating the volumetric load of the LA, they may be insufficient to fully reflect its complex mechanical function. To address this limitation, new parameters that combine both the volumetric load and the contractile function of the LA are being developed. The Left Atrial Coupling Index (LACI) is a relatively novel echocardiographic marker that reflects the mechanical–volumetric coupling of the LA, calculated as the ratio of left atrial volume index (LAVI) to the atrial contraction velocity (a′ wave) measured by tissue Doppler imaging (TDI).

LACI has been described in the literature; however, no study has evaluated its predictive value for POAF following isolated CABG. The aim of the present study was to investigate the predictive role of preoperatively measured LACI values for POAF following isolated CABG and to compare two different calculation methods, referred to as LACI_1_ and LACI_2_. We hypothesized that preoperative LACI values would predict the development of POAF following isolated CABG and that the novel calculation method (LACI_2_) might provide better predictive value compared with the conventional method (LACI_1_).

## 2. Materials and Methods

### 2.1. Study Design and Population

This prospective study was conducted between August 2023 and May 2024 in 133 patients who were scheduled to undergo CABG in our clinic. Three patients were excluded because they declined to undergo surgery, leaving a final study population of 130 patients. The exclusion criteria were as follows:

- Patients younger than 18 years or older than 80 years.
- Patients with a prior diagnosis of AF or who developed preoperative AF during hospitalization.
- Patients with congenital valvular disease or valvular pathology requiring surgical intervention.

All included patients underwent preoperative transthoracic echocardiography, and LACI values were calculated. Patients were then categorized into two groups according to the development of POAF: POAF (−) and POAF (+). LACI values, along with other demographic, clinical, and echocardiographic parameters, were statistically compared between the two groups.

Demographic, clinical, laboratory, and echocardiographic data were recorded for all patients preoperatively. The study protocol was approved by the institutional ethics committee, and written informed consent was obtained from all participants.

### 2.2. Echocardiographic Assessment and LACI Measurements

Preoperative transthoracic echocardiographic examinations were performed in all patients using a Philips EPIQ 7 system (Philips Healthcare, Amsterdam, The Netherlands) equipped with a 2.5–3.5 MHz transducer. Standard echocardiographic parameters were measured in accordance with the recommendations of the American Society of Echocardiography [15]. All echocardiographic assessments were conducted by the same experienced specialist to minimize interobserver variability. LAVI was calculated using the biplane area–length method from apical four- and two-chamber views and was indexed to body surface area (BSA).

LACI was calculated using the following formulas:

- LACI_1_ = LAVI/TDI–septal a′ (cm/s)
- LACI_2_ = LAVI/min[TDI–septal a′, TDI–lateral a′] (cm/s)

In these formulas, the TDI–a′ wave represents the peak velocity during atrial systole obtained by tissue Doppler imaging from the septal and lateral mitral annulus. The term min[TDI–septal a′, TDI–lateral a′] refers to the lowest value between the septal and lateral a′ waves.

Although the LACI formula based on LAVI and TDI–septal a′ has been described in the literature, in the present study, we refer to this conventional formula as LACI_1_. The LACI_2_ formula, which incorporates the lower of the septal and lateral a′ velocities, is introduced here as a novel parameter that has not been previously reported.

### 2.3. Postoperative Follow-Up and Diagnostic Criteria

All patients were monitored postoperatively in the intensive care unit and subsequently on the ward, with continuous cardiac rhythm surveillance via telemetry. POAF was defined as new-onset AF occurring in the postoperative period, documented by electrocardiography (ECG) or telemetry, and characterized by the absence of discernible P waves and an irregular R–R interval. On telemetry, the diagnosis was made regardless of episode duration.

### 2.4. Statistical Analysis

Statistical analyses were performed using SPSS software, version 29.0.2.0 (IBM Corp., Armonk, NY, USA). Continuous variables were expressed as mean ± standard deviation, and categorical variables were expressed as counts and percentages. The Kolmogorov–Smirnov test was used to assess the normality of data distribution. Normally distributed continuous variables were compared using Student’s *t*-test, whereas non-normally distributed variables were compared using the Mann–Whitney *U* test. Associations between categorical variables were evaluated using the chi-square test or Fisher’s exact test, as appropriate. Logistic regression analysis was performed to identify independent predictors of POAF. Receiver operating characteristic (ROC) curve analysis was used to determine the optimal cut-off value for LACI, and the sensitivity, specificity, positive predictive value (PPV), and negative predictive value (NPV) corresponding to this cut-off were calculated. All statistical tests were two-tailed, and a *p*-value of <0.05 was considered statistically significant. Comparisons of ROC curves of the inflammation-based scores and determination of cut-off value were performed using the MEDCALC software, version 23.4.5 program (MedCalc Software bv, Ostend, Belgium).

## 3. Results

A total of 130 patients who underwent isolated CABG were included in the present study. POAF occurred in 59 patients (45.4%), whereas 71 patients (54.6%) did not develop POAF.

### 3.1. Patient Demographic and Clinical Characteristics

The mean age of the patients with POAF was significantly higher than that of those who did not develop POAF (63.02 ± 9.55 vs. 59.45 ± 8.53, respectively; *p* = 0.023). Body mass index (BMI) also differed significantly between the groups (*p* = 0.03). The prevalence of diabetes mellitus (DM) (*p* = 0.011) and hypertension (HT) (*p* = 0.027) was significantly higher in the POAF group. In addition, a significant association was found between POAF occurrence and EuroSCORE II values (*p* < 0.001) (Table 1).

No statistically significant differences were observed between the groups in terms of sex (*p* = 0.872), history of stroke (*p* = 0.296), or smoking status (*p* = 0.437) (Table 1). Similarly, there was no significant association between the number of grafts used during surgery and the development of POAF (*p* = 0.880).

### 3.2. Clinical Outcomes

The development of POAF was significantly associated with length of hospital stay andin-hospital mortality. The length of hospital stay was longer in patients who developed POAF compared to those without POAF (15.98 ± 16.29 days vs. 11.07 ± 21.84 days, *p* < 0.001). The in-hospital mortality rate was 13.5% in the POAF group and 24.2% in the non-POAF group (*p* = 0.014) (Table 2).

### 3.3. Echocardiographic Findings

LA length was significantly greater in patients who developed POAF (38.57 ± 4.02 mm) than those who did not develop POAF (36.72 ± 3.86 mm, *p* = 0.018). Left ventricular ejection fraction (EF) and LAVI values showed no significant differences between the groups (*p* = 0.108 and *p* = 0.249, respectively). In the subgroup analysis stratified by impaired EF (<50%) and preserved EF (≥50%), no statistically significant difference was observed between the two groups. Among the tissue Doppler parameters, TDI septal a′, TDI lateral e′, and TDI lateral a′ values were found to be significantly lower in the POAF group compared to the non-POAF group (*p* = 0.02, *p* = 0.048, and *p* < 0.001, respectively) (Table 3).

### 3.4. Analysis of LACI and ROC Curve Results

LACI, calculated as the ratio of LAVI to TDI-a′, was analyzed using two different methods.

- LACI1: The mean value was 4.21 ± 2.62 in the POAF group and 2.94 ± 1.02 in the non-POAF group, showing a statistically significant difference (*p* < 0.001).
- LACI2: The mean value was 4.27 ± 2.60 in the POAF group and 2.96 ± 1.02 in the non-POAF group, also demonstrating a statistically significant difference (*p* < 0.001).

**Figure 1:**
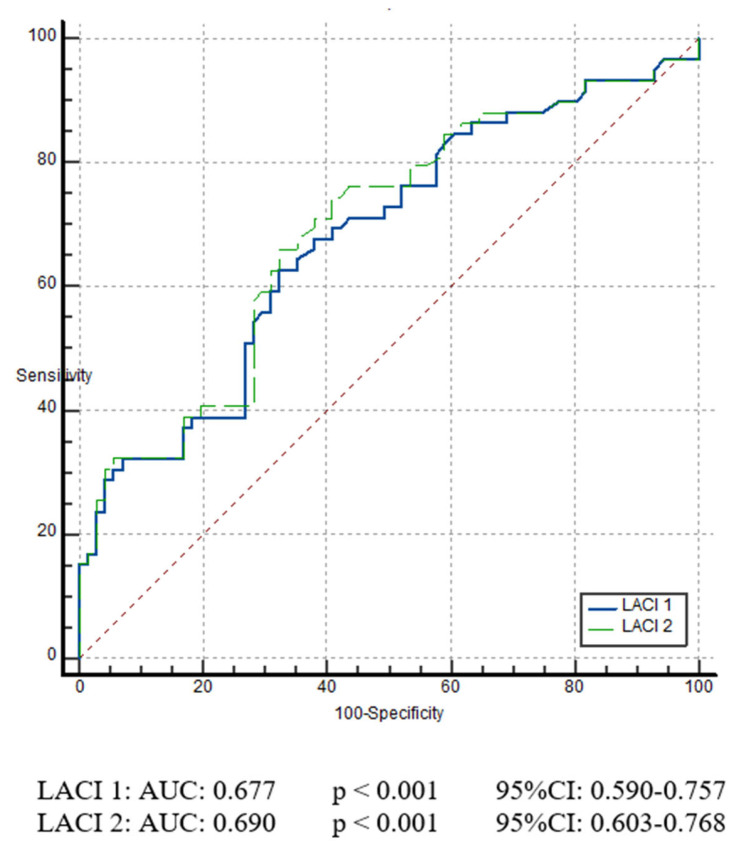
Receiver operating characteristic (ROC) curves of LACI_1_ and LACI_2_ for predicting POAF. Abbreviations: AUC, area under the curve; LACI, left atrial coupling index; *p*, *p*-value; 95% CI, 95% confidence interval.

Due to the ROC analysis that was performed to evaluate the predictive ability of LACI_1_ and LACI_2_ for the development of POAF, LACI_2_ demonstrated better predictive performance compared with LACI_1_. For LACI_1_, the area under the curve (AUC) was 0.677 (*p* < 0.001), with a sensitivity of 62.71% and a specificity of 67.6%. For LACI_2_, the AUC was 0.690 (*p* < 0.001), with a sensitivity of 59.1% and a specificity of 74.5% (Figure 1). Although the AUC of LACI_2_ was numerically higher than that of LACI_1_, the difference did not reach statistical significance on DeLong testing (ΔAUC = 0.012; *p* = 0.149).

Univariate logistic regression analysis revealed that age (*p* = 0.033), DM (*p* = 0.012), HT (*p* = 0.028), EuroScore II (*p* = 0.013), LA length (*p* = 0.023), TDI-lateral e′ (*p* = 0.021), TDI-lateral a′ (*p* = 0.014), LAVI (*p* = 0.048), LACI_1_ (*p* < 0.001), and LACI_2_ (*p* < 0.001) were significantly associated with the development of POAF. In addition, in-hospital mortality was also found to be significantly higher in the POAF group in the univariate analysis (Table 4).

Multivariate logistic regression analysis revealed that LACI_1_ (*p* = 0.020, OR: 1.450) and LACI_2_ (*p* = 0.004, OR: 1.503) remained independent predictors of POAF (Table 5).

In these analyses, EuroSCORE II was excluded from the multivariate logistic regression to avoid multicollinearity, as it already incorporates age and DM as components. Similarly, LAVI and LA length were excluded to prevent multicollinearity because they are integral components of the LACI parameter, which was included in the analysis.

## 4. Discussion

In this prospective study, the prognostic and predictive value of the preoperatively calculated LACI parameter for POAF was investigated in patients who underwent isolated CABG. The main findings of our study were as follows:

1. Both LACI_1_ and LACI_2_—of which LACI_2_ has not been previously reported in the literature—were found to be independent and significant predictors of POAF after isolated CABG.
2. LACI_2_ demonstrated a stronger predictive value compared with LACI_1_.
3. LACI was shown to be a superior predictor compared with other conventional LA parameters, such as LAVI and LA length.
4. Our findings also support the conclusion that POAF was significantly associated with prolonged hospitalization and increased in-hospital mortality.

AF is a complex arrhythmia with multiple underlying mechanisms. Structural heart disease can trigger atrial remodeling, leading to dissociation between myofibrils and electrical conduction, thereby facilitating both the initiation and maintenance of the arrhythmia [16,17]. In addition to these structural alterations, inflammation and oxidative stress contribute to endothelial activation and injury, promote fibrinogen formation, and induce fibrotic changes [1]. Within this pathophysiological framework, the functional status of the LA is closely associated with several pathological conditions, including valvular heart disease, heart failure, and arrhythmias, particularly AF and POAF [18].

Numerous preoperative risk factors for predicting POAF following CABG have been identified in the literature, including advanced age, HT, DM, congestive heart failure, obesity, and higher EuroSCORE II values [19]. The findings of our study are consistent with previous reports, confirming the association between these parameters and the development of POAF. In contrast to prior reports, left ventricular EF was not significantly associated with POAF in our cohort. Similarly, no significant difference was observed when patients were categorized into impaired and preserved EF subgroups (Table 3). Although the mechanisms underlying AF and POAF largely overlap, distinct pathophysiological pathways may also exist. In this context, the lack of a statistically significant association between EF and POAF in our study may, at least in part, be attributable to the relatively limited sample size.

Perioperative and postoperative stress contribute to remodeling of the atrial substrate. Hence, surgery provokes two essential arrhythmogenic mechanisms within the atrium: triggered activity and re-entry [11,20]. In this context, assessing the relationship between the atrium and the development of AF solely by size or volume is inadequate. Supporting this notion, Rostagno et al. reported that LA size was not significantly associated with AF recurrence in patients with paroxysmal lone AF [21]. Although a meta-analysis has demonstrated LAVI to be a predictor of POAF after cardiac surgery [22], in some cohorts—such as the study authored by Her et al. [23] and the CABG cohort reported by Başaran et al. [24], which evaluated LA and ventricular function—LAVI lost statistical significance in multivariable logistic regression. While the value of LAVI is undeniable, it reflects only the volumetric aspect of LA remodeling. By contrast, LACI integrates both volumetric and mechanical/contractile components of LA function and is, therefore, a theoretically more comprehensive marker of atrial pathology. Consistent with this concept, our data show that LACI outperforms LAVI in predicting POAF after isolated CABG.

Despite these findings, LAVI reflects only the volumetric aspect of LA remodeling, whereas LACI integrates both volumetric and mechanical/contractile components, providing a more comprehensive index of LA function. In our cohort, LAVI showed no significant difference between POAF groups on unadjusted comparisons (*p* = 0.249), yet demonstrated a borderline trend in univariate logistic regression. In contrast, LACI1 (*p* < 0.001) and LACI2 (*p* < 0.001) were more strongly associated with POAF, and this association remained significant in both univariate and multivariable logistic regression analyses, suggesting that LACI may better capture volumetric–mechanical coupling implicated in POAF pathogenesis.

Previous studies have evaluated LACI in non-surgical cohorts: Benfari et al. (2021) linked LACI to adverse outcomes in patients with reduced ejection fraction, and Essayagh et al. (2022) demonstrated its prognostic value in mitral valve prolapse [25,26]. In our study, we referred to this previously described parameter as LACI_1_ and introduced a modified version, LACI2, which has not been reported in the literature. Under normal circumstances, the TDI-lateral a′ value is greater than the TDI-septal a′ value. However, certain conditions, such as storage diseases or left ventricular wall motion abnormalities, may alter this pattern and affect the LACI calculation. For example, in a patient with lateral wall akinesia, the TDI-lateral a′ value may be lower than the TDI-septal a′ value. Since such conditions can affect measurement accuracy, we proposed the LACI2 formula to enhance the predictive value of our assessment. In daily cardiology practice, LACI_1_ and LACI_2_ can be easily calculated using standard transthoracic echocardiography, making them cost-effective and widely available parameters. Importantly, LACI_2_ may be incorporated into routine preoperative risk stratification to identify patients at higher risk of POAF, thereby supporting individualized monitoring and preventive strategies alongside established clinical risk factors.

A key contribution of our study is that LACI_2_, which has not been evaluated previously, outperformed LACI_1_. LACI_2_ yielded an AUC of 0.690 versus 0.677 for LACI_1_, and a higher specificity (74.5% vs. 67.6%). Although the difference between AUCs did not reach statistical significance, the consistently higher AUC observed for LACI_2_ may indicate a potential incremental discriminatory ability. In this context, despite the modest absolute AUC difference, incorporating min(a′) may better reflect the weakest atrial contraction segment, offering a more representative assessment of global LA functional integrity and potentially refining risk stratification, a hypothesis that requires validation in larger, adequately powered cohorts.

In our study, the rate of POAF was relatively higher compared to previous reports [12]. The primary reason for this finding is that, unlike many earlier studies, we did not set a minimum duration threshold for AF; POAF was defined as the occurrence of AF at any time, regardless of its duration. Consistent with this approach, Tsai et al. also reported a high incidence of POAF in patients undergoing isolated CABG, which may partly reflect differences in the definitions and diagnostic criteria used for AF, like in our study [27]. Consistent with prior literature, POAF in our cohort was associated with longer length of stay and higher in-hospital mortality. As a preoperatively accessible metric, LACI may therefore help predict not only arrhythmia risk but also clinically meaningful outcomes linked to POAF, enabling proactive perioperative strategies (e.g., beta-blockers, amiodarone prophylaxis) in high-risk patients. With the increasing incidence of AF, substantial advances have also been achieved in the treatment of AF. In patients with pre-existing AF undergoing CABG, surgical AF ablation [28] represents an effective therapeutic strategy in this context. In parallel with advances in AF treatment, electrophysiology practice has entered a new era characterized by radiation-free strategies. Zero-fluoroscopy ablation approaches guided by three-dimensional electroanatomical mapping have been shown to be feasible and safe, substantially reducing radiation exposure for both patients and operators [29]. If preoperative predictors of POAF can be identified with high specificity and integrated into a refined risk stratification algorithm, selected high-risk patients may be candidates for prophylactic surgical AF ablation [28] as a preventive strategy against POAF, which may help reduce the morbidity and mortality associated with POAF.

### Limitations

This is a single-center study with a modest sample size, which may limit generalizability. In addition, intraobserver variability was not assessed during the echocardiographic study. External validation across broader populations and other cardiac surgeries is also warranted. Further work is also needed to facilitate the routine clinical implementation of LACI_2_.

## 5. Conclusions

Preoperative LACI is an independent and robust predictor of POAF after isolated CABG. Notably, LACI_2_—despite being novel—showed relatively better predictive performance than LACI_1_. Incorporating LACI into preoperative assessment may aid in identifying high-risk patients and guiding strategies to reduce POAF-related morbidity and mortality.

## Figures and Tables

**Table 1:** Demographic and Clinical Characteristics.

Variable	POAF (−) (n =71)	POAF (+) (n = 59)	*p*-Value
Age (years)	59.45 ± 8.53	63.02 ± 9.55	0.023
Body mass index (BMI), kg/m^2^	27.95 ± 5.43	29.25 ± 4.68	0.030
Height, cm	168.00 ± 9.09	165.00 ± 9.05	0.810
Weight, kg	79.66 ± 16.38	80.24 ± 14.07	0.540
Body surface area (BSA), m^2^	1.92 ± 0.21	1.91 ± 0.19	0.820
Sex, male/female, n (%)	55 (42)/16 (12)	45 (35)/14 (11)	0.872
ACS/CCS, n	35/36	30/29	0.625
Diabetes mellitus (DM), n (%)	31 (43.6)	37 (64.9)	0.017
Hypertension (HT), n (%)	39 (54.9)	42 (71.2)	0.029
Prior stroke, n (%)	4 (5.6)	6 (10.1)	0.296
EuroSCORE II	1.17 ± 0.85	1.89 ± 1.51	<0.001
Current smoker, n (%)	26 (36.6)	17 (28.8)	0.437

Abbreviations: ACS, acute coronary Syndrome; CCS, chronic coronary syndrome.

**Table 2:** Comparison of Perioperative Characteristics and in-Hospital Outcomes Between POAF and Non-POAF Groups.

Variable	POAF (−) (n = 71)	POAF (+) (n = 59)	*p*-Value
In-hospital mortality, n (%)	3 (4.2)	8 (13.5)	0.014
Length of hospital stay, days	11.07 ± 21.84	15.98 ± 16.29	<0.001
Operation time, min	292.16 ± 45.99	305.56 ± 59.15	0.201

**Table 3:** Comparison of Echocardiographic Parameters Between POAF and Non-POAF Groups.

Parameter	POAF (−) (n = 71)	POAF (+) (n = 59)	*p*-Value
EF (%)	56.51 ± 6.44	54.03 ± 8.33	0.108
EF < 50%, n (%)	12 (9)	16 (12)	0.090
EF ≥ 50%, n (%)	59 (46)	43 (33)	
Interventricular septum thickness, mm	10.73 ± 1.49	11.07 ± 2.16	0.510
Posterior wall thickness, mm	9.89 ± 1.30	9.98 ± 2.22	0.864
Left atrial diameter, mm	36.72 ± 3.86	38.57 ± 4.02	0.018
E wave velocity, cm/s	89.33 ± 19.58	92.60 ± 22.63	0.147
A wave velocity, cm/s	88.07 ± 21.52	97.10 ± 21.73	0.490
E/A ratio	1.07 ± 0.37	1.02 ± 0.44	0.235
TDI–septal e′, cm/s	7.86 ± 2.52	7.14 ± 2.03	0.134
TDI–septal a′, cm/s	10.92 ± 1.79	9.76 ± 2.48	0.020
TDI–lateral e′, cm/s	9.96 ± 2.93	8.76 ± 2.60	0.048
TDI–lateral a′, cm/s	12.98 ± 1.60	11.77 ± 3.38	<0.001
Pulmonary artery systolic pressure (PASP), mmHg	26.68 ± 7.12	30.75 ± 8.40	0.248
Left atrial volume index (LAVI), mL/m^2^	31.81 ± 9.39	35.38 ± 10.63	0.249

Abbreviations: POAF, postoperative atrial fibrillation; EF, ejection fraction; TDI, tissue Doppler imaging.

**Table 4:** Univariate Logistic Regression Analysis.

Variable	*p*-Value	OR	95% CI
Age	0.033	1.045	1.004–1.088
BMI	0.157	1.054	0.980–1.133
DM	0.012	2.498	1.221–5.110
HT	0.028	2.304	1.094–4.854
EuroScore II	0.013	1.957	1.154–3.317
Left atrial length	0.023	1.131	1.017–1.257
TDI-Septal e′	0.086	0.869	0.740–1.020
TDI-Septal a′	0.004	0.772	0.646–0.922
TDI-Lateral e′	0.021	0.851	0.742–0.976
TDI-Lateral a′	0.014	0.832	0.719–0.964
LAVI	0.048	1.037	1.001–1.075
LACI_1_	<0.001	1.665	1.237–2.242
LACI_2_	<0.001	1.723	1.273–2.333
In-hospital mortality	0.023	4.673	1.238–17.638
Operation time	0.154	1.005	0.998–1.012

Abbreviations: BMI, body mass index; DM, diabetes mellitus; HT, hypertension; TDI, tissue Doppler imaging; LAVI, left atrial volume index; LACI, left atrial coupling index; OR, odds ratio; 95% CI, 95% confidence interval.

**Table 5:** a: Model including LACI_1_; b: Model including LACI_2_.

Variable	*p*-Value	OR	95% CI
(**a**)			
Age	0.273	1.027	0.979–1.077
DM	0.084	0.491	0.219–1.100
HT	0.340	0.667	0.287–1.538
LACI_1_	0.020	1.450	1.060–1.983
TDI-Lateral e′	0.388	0.931	0.792–1.093
(**b**)			
Age	0.282	1.027	0.979–1.077
DM	0.081	0.487	0.217–1.094
HT	0.344	0.667	0.288–1.545
LACI_2_	0.004	1.503	1.091–2.072
TDI-Lateral e′	0.434	0.937	0.796–1.103

Abbreviations: DM, diabetes mellitus; HT, hypertension; TDI, tissue Doppler imaging; LACI, left atrial coupling index; OR, odds ratio; 95% CI, 95% confidence interval.

## Data Availability

The original contributions presented in this study are included in the article. Further inquiries can be directed to the corresponding author.

## References

[1] Magnussen C., Niiranen T.J., Ojeda F.M., Gianfagna F., Blankenberg S., Njolstad I., Vartiainen E., Sans S., Pasterkamp G., Hughes M. (2017). Sex Differences and Similarities in Atrial Fibrillation Epidemiology, Risk Factors, and Mortality in Community Cohorts: Results From the BiomarCaRE Consortium (Biomarker for Cardiovascular Risk Assessment in Europe). Circulation.

[2] Mont L., Elosua R., Brugada J. (2009). Endurance sport practice as a risk factor for atrial fibrillation and atrial flutter. Europace.

[3] Bizhanov K.A., Abzaliyev K.B., Baimbetov A.K., Sarsenbayeva A.B., Lyan E. (2023). Atrial fibrillation: Epidemiology, pathophysiology, and clinical complications (literature review). J. Cardiovasc. Electrophysiol..

[4] Schnabel R.B., Yin X., Gona P., Larson M.G., Beiser A.S., McManus D.D., Newton-Cheh C., Lubitz S.A., Magnani J.W., Ellinor P.T. (2015). 50 year trends in atrial fibrillation prevalence, incidence, risk factors, and mortality in the Framingham Heart Study: A cohort study. Lancet.

[5] Frost L., Vestergaard P., Mosekilde L. (2004). Hyperthyroidism and risk of atrial fibrillation or flutter: A population-based study. Arch. Intern. Med..

[6] Chamberlain A.M., Agarwal S.K., Folsom A.R., Duval S., Soliman E.Z., Ambrose M., Eberly L.E., Alonso A. (2011). Smoking and incidence of atrial fibrillation: Results from the Atherosclerosis Risk in Communities (ARIC) study. Heart Rhythm..

[7] Buckley B.J.R., Harrison S.L., Gupta D., Fazio-Eynullayeva E., Underhill P., Lip G.Y.H. (2021). Atrial Fibrillation in Patients With Cardiomyopathy: Prevalence and Clinical Outcomes From Real-World Data. J. Am. Heart Assoc..

[8] Lubitz S.A., Yin X., Rienstra M., Schnabel R.B., Walkey A.J., Magnani J.W., Rahman F., McManus D.D., Tadros T.M., Levy D. (2015). Long-term outcomes of secondary atrial fibrillation in the community: The Framingham Heart Study. Circulation.

[9] Gaudino M., Di Franco A., Rong L.Q., Piccini J., Mack M. (2023). Postoperative atrial fibrillation: From mechanisms to treatment. Eur. Heart J..

[10] Greenberg J.W., Lancaster T.S., Schuessler R.B., Melby S.J. (2017). Postoperative atrial fibrillation following cardiac surgery: A persistent complication. Eur. J. Cardiothorac. Surg..

[11] Mathew J.P., Fontes M.L., Tudor I.C., Ramsay J., Duke P., Mazer C.D., Barash P.G., Hsu P.H., Mangano D.T., Investigators of the Ischemia Research and Education Foundation and the Multicenter Study of Perioperative Ischemia Research Group (2004). A multicenter risk index for atrial fibrillation after cardiac surgery. JAMA.

[12] Eikelboom R., Sanjanwala R., Le M.L., Yamashita M.H., Arora R.C. (2021). Postoperative Atrial Fibrillation After Cardiac Surgery: A Systematic Review and Meta-Analysis. Ann. Thorac. Surg..

[13] Haemers P., Hamdi H., Guedj K., Suffee N., Farahmand P., Popovic N., Claus P., LePrince P., Nicoletti A., Jalife J. (2017). Atrial fibrillation is associated with the fibrotic remodelling of adipose tissue in the subepicardium of human and sheep atria. Eur. Heart J..

[14] Lee S.H., Kang D.R., Uhm J.S., Shim J., Sung J.H., Kim J.Y., Pak H.N., Lee M.H., Joung B. (2014). New-onset atrial fibrillation predicts long-term newly developed atrial fibrillation after coronary artery bypass graft. Am. Heart J..

[15] Mitchell C., Rahko P.S., Blauwet L.A., Canaday B., Finstuen J.A., Foster M.C., Horton K., Ogunyankin K.O., Palma R.A., Velazquez E.J. (2019). Guidelines for Performing a Comprehensive Transthoracic Echocardiographic Examination in Adults: Recommendations from the American Society of Echocardiography. J. Am. Soc. Echocardiogr..

[16] Bailey G.W., Braniff B.A., Hancock E.W., Cohn K.E. (1968). Relation of left atrial pathology to atrial fibrillation in mitral valvular disease. Ann. Intern. Med..

[17] John B., Stiles M.K., Kuklik P., Chandy S.T., Young G.D., Mackenzie L., Szumowski L., Joseph G., Jose J., Worthley S.G. (2008). Electrical remodelling of the left and right atria due to rheumatic mitral stenosis. Eur. Heart J..

[18] Guo Y., Lip G.Y., Apostolakis S. (2012). Inflammation in atrial fibrillation. J. Am. Coll. Cardiol..

[19] Dobrev D., Aguilar M., Heijman J., Guichard J.B., Nattel S. (2019). Postoperative atrial fibrillation: Mechanisms, manifestations and management. Nat. Rev. Cardiol..

[20] Bhave P.D., Goldman L.E., Vittinghoff E., Maselli J., Auerbach A. (2012). Incidence, predictors, and outcomes associated with postoperative atrial fibrillation after major noncardiac surgery. Am. Heart J..

[21] Rostagno C., Olivo G., Comeglio M., Bertini G., Gensini G.F., Galanti G. (1996). Left atrial size changes in patients with paroxysmal lone atrial fibrillation. An echocardiographic follow-up. Angiology.

[22] Kirchhof P., Benussi S., Kotecha D., Ahlsson A., Atar D., Casadei B., Castella M., Diener H.C., Heidbuchel H., Hendriks J. (2016). 2016 ESC Guidelines for the management of atrial fibrillation developed in collaboration with EACTS. Europace.

[23] Her A.Y., Kim J.Y., Kim Y.H., Choi E.Y., Min P.K., Yoon Y.W., Lee B.K., Hong B.K., Rim S.J., Kwon H.M. (2013). Left atrial strain assessed by speckle tracking imaging is related to new-onset atrial fibrillation after coronary artery bypass grafting. Can. J. Cardiol..

[24] Başaran Ö., Tigen K., Gözübüyük G., Dündar C., Güler A., Taşar O., Biteker M., Karabay C.Y., Bulut M., Karaahmet T. (2016). Predictive role of left atrial and ventricular mechanical function in postoperative atrial fibrillation: A two-dimensional speckle-tracking echocardiography study. Turk. Kardiyol. Dern. Ars..

[25] Essayagh B., Benfari G., Antoine C., Maalouf J., Pislaru S., Thapa P., Michelena H.I., Enriquez-Sarano M. (2022). Incremental Prognosis by Left Atrial Functional Assessment: The Left Atrial Coupling Index in Patients With Floppy Mitral Valves. J. Am. Heart Assoc..

[26] Benfari G., Essayagh B., Nistri S., Maalouf J., Rossi A., Thapa P., Michelena H.I., Enriquez-Sarano M. (2021). Left Atrial Volumetric/Mechanical Coupling Index: A Novel Predictor of Outcome in Heart Failure With Reduced Ejection Fraction. Circ. Cardiovasc. Imaging.

[27] Tsai Y.T., Lai C.H., Loh S.H., Lin C.Y., Lin Y.C., Lee C.Y., Ke H.Y., Tsai C.S. (2015). Assessment of the Risk Factors and Outcomes for Postoperative Atrial Fibrillation Patients Undergoing Isolated Coronary Artery Bypass Grafting. Acta Cardiol. Sin..

[28] Nisivaco S., Lysyy T., Kruse J., Cox J.L., Malaisrie S.C. (2025). Surgical treatment of atrial fibrillation in coronary artery bypass grafting. J. Thorac. Cardiovasc. Surg..

[29] Mascia G., Giaccardi M. (2020). A New Era in Zero X-ray Ablation. Arrhythm. Electrophysiol. Rev..

